# One‐step selective affinity purification and immobilization of His‐tagged enzyme by recyclable magnetic nanoparticles

**DOI:** 10.1002/elsc.202000093

**Published:** 2021-05-04

**Authors:** Li‐Jian Zhou, Rui‐Fang Li, Xue‐Yong Li, Ye‐Wang Zhang

**Affiliations:** ^1^ The People's Hospital of Danyang Affiliated Danyang Hospital of Nantong University Danyang Jiangsu Province P. R. China; ^2^ School of Pharmacy Jiangsu University Zhenjiang P. R. China

**Keywords:** glucose dehydrogenase, His‐tagged recombinant protein, immobilization, purification

## Abstract

The NiFe_2_O_4_ magnetic nanoparticles (NF‐MNPs) were prepared for one‐step selective affinity purification and immobilization of His‐tagged recombinant glucose dehydrogenase (GluDH). The prepared nanoparticles were characterized by a Fourier‐transform infrared spectrophotometer and microscopy. The immobilization and purification of His‐tagged GluDH on NF‐MNPs were investigated. The optimal immobilization conditions were obtained that mixed cell lysis and carriers in a ratio of 0.13 in pH 8.0 Tris‐HCl buffer at 30℃ and incubated for 2 h. The highest activity recovery and protein bindings were 71.39% and 38.50 μg mg^–1^ support, respectively. The immobilized GluDH exhibited high thermostability, pH‐stability and it can retain more than 65% of the initial enzyme after 10 cycles for the conversion of glucose to gluconolactone. Comparing with a commercial Ni‐NTA resin, the NF‐MNPs displayed a higher specific affinity with His‐tagged recombinant GluDH.

**Abbreviations**
DNS: 3,5‐dinitrosalicylic acid
GluDH: glucose dehydrogenase
NF‐MNPs: NiFe_2_O_4_ magnetic nanoparticles
NTA: nitrogen triacetic acid

## 1 INTRODUCTION

The separation and purification of protein are one of the key steps in the industrial production of proteins. The traditional methods are hydrophobic chromatography [1], ultrafiltration [2], ion‐exchange chromatography [3], and gel filtration chromatography [4]. The methods are chosen according to the properties of the protein and the requirements of the separation. Furthermore, protein tags also play an essential role in the purification of the recombinant protein. Due to the relatively small molecular weight, low immunogenicity, hydrophilicity, hexahistidine is recognized as the most widely used affinity label [5, 6, 7, 8]. The imidazole group on the hexahistidine can be used to specifically interact with transition metal ions for a series of protein purification and immobilization purposes. Based on the immobilized metal ion affinity chromatography (IMAC) proposed by Porath et al. [9] Researchers have paid much effort to promote the development of protein purification and immobilization up to now. At present, several materials have been introduced to purify His‐tagged protein. Metal‐resin complex is mostly obtained support by fixing metal ions on resin microspheres through complex agents, such as nitrogen triacetic acid (NTA) [10, 11], or iminodiacetic acid (IDA) [12, 13]. However, these materials have some drawbacks like time‐consuming operation, complicated pretreatment, poor mechanical stability [14, 15]. Metal‐protein hybrid is also used for the immobilization of including his‐tagged enzymes [16, 17], which was developed by encapsulating enzymes using metal components. However, the synthesis of metal‐protein hybrid is tedious, and the productivity is quite low [18]. Other nanoparticles, such as metal‐organic framework (MOF) [19], magnetic nanoparticle [20, 21, 22] also be employed for enzyme immobilization. The drawback of metal ion derivative nanoparticles is that the metal ions are easily leaked during use. Among these materials, the magnetic nanoparticles exhibit the advantage of easy separation over non‐magnetic particles from the reaction mixture with an external magnetic field [23, 24, 25]. However, nickel ferrite, a kind of easily prepared and separated magnetic spinel ferrite nanoparticle, can be used as support for one‐step immobilization and purification of His‐tagged protein avoiding the above problems. Besides, spinel ferrites have attracted significant attention on account of their fascinating magnetic and electromagnetic properties [12]. Among spinel ferrites, nickel ferrite (NiFe_2_O_4_) is a soft magnetic material with low coercivity, high saturation magnetization, chemical stability, and electrical resistivity [26, 27]. Hence, it is suitable for several technological applications such as electrochemical sensors [28, 29, 30], supercapacitor [31], adsorbent [32, 33, 34].

Glucose dehydrogenase (EC1.1.1.47, GluDH), one of the short‐chain dehydrogenase superfamily, is a tetramer protein consisting of four subunits [35]. It mainly was used for specifically conversion from β‐d‐glucose to β‐d‐gluconolactone using NAD(P)^+^ as a cofactor, and also can be used in diagnostic for clinical blood glucose measurement [36, 37], electrochemical detection [38], as well as biofuel cell [39, 40]. Besides, it can be employed for cofactor regeneration [41]. Despite the wide range of applications and interests, the free glucose dehydrogenase has some inevitable limitations such as complex purification process, unrecyclable, and difficult to separate from product. The one‐step immobilization and purification enzyme using recyclable magnetic nanoparticles can work out these problems.

**PRACTICAL APPLICATION**

One‐step purification and immobilization of the recombinant hexahistidine tagged glucose dehydrogenase (GluDH) was realized with prepared NiFe_2_O_4_ magnetic nanoparticles (NF‐MNPs). The magnetic nanoparticles were prepared with a simple hydrothermal method. After sonication of the cultured cells, the supernatant containing GluDH was obtained by centrifugation of the cell debris; then the nanoparticles were added directly for purification and immobilization. The NF‐MNPs displayed high binding specificity to hexahistidine tagged GluDH. The immobilized GluDH showed enhanced stability and reusability. The thermostability and pH‐stability of the immobilized GluDH were 3.86 times and 27 times as much as that of free GluDH. The affinity between NF‐MNPs and His‐tagged GluDH was so highly specific that only the GluDH was detected in the eluent with the elution by imidazole. The NF‐MNPs might have promising applications in the purification and immobilization of other hexahistidine tagged recombinant proteins.

**HIGHLIGHTS**

- Nickel ferrites were prepared and displayed high specific binding of His‐tagged glucose dehydrogenase.
- One‐step purification and immobilization of the glucose dehydrogenase were achieved.
- The immobilized glucose dehydrogenase exhibited excellent reusability and stability.

**FIGURE 1:**
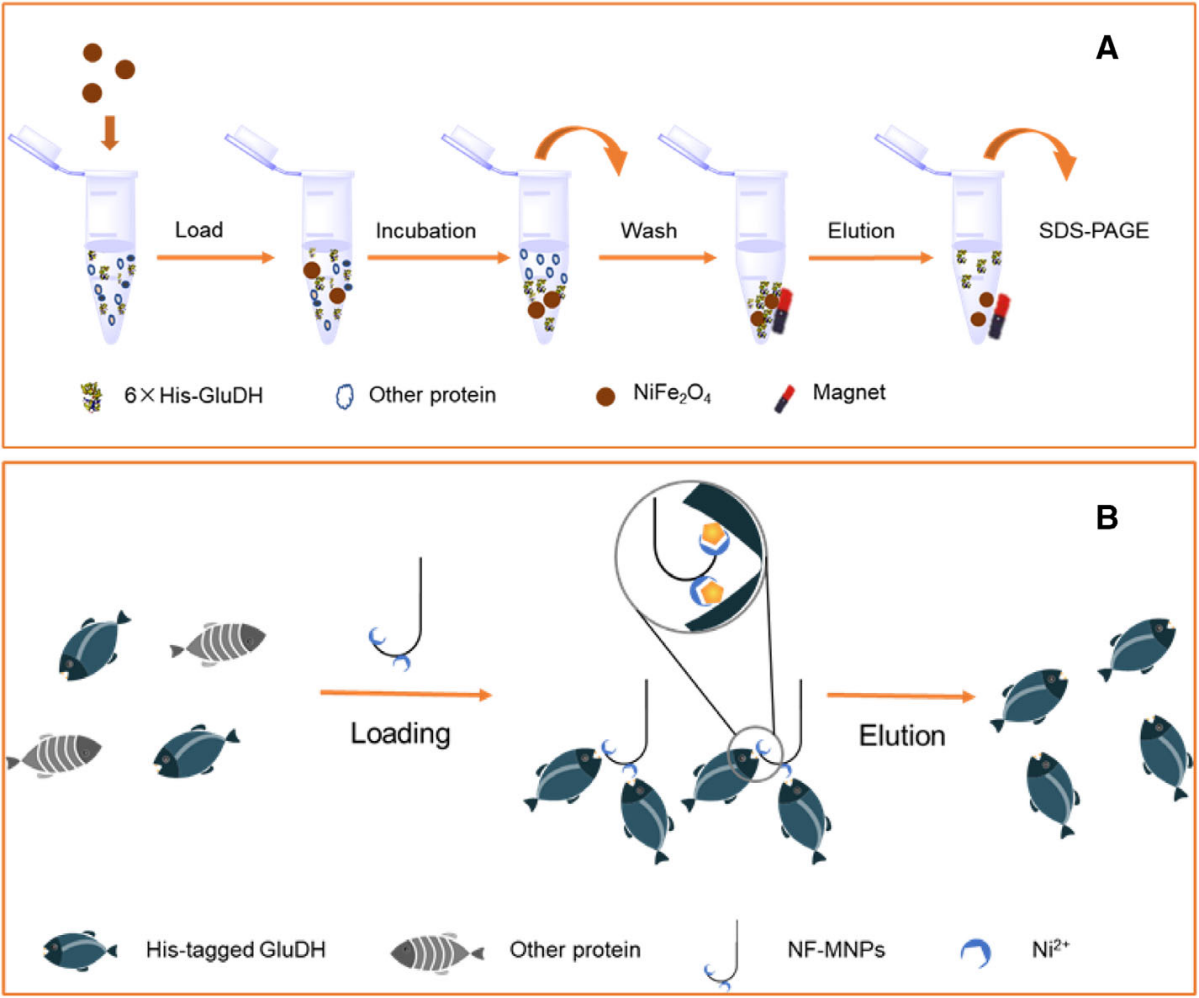
Scheme of one‐step immobilization and purification of His‐tagged GluDH on NiFe_2_O_4_ magnetic nanoparticles. (A) The process for purification of GluDH. (B) Illustration of specific immobilization and purification of GluDH

In this work, the superparamagnetic and recyclable nickel ferrites were prepared and characterized. One‐step selective affinity purification and immobilization of His‐tagged recombinant GluDH was achieved from crude cell lysis through the IMAC method (as shown in Figure 1). Furthermore, the purification of GluDH showed high selectivity, while the immobilization improved the enzyme's stability and practical reusability.

## 2 MATERIALS AND METHODS

### 2.1 Materials

NaCl, yeast extract, Tryptone, agar, imidazole, d‐glucose, Nicotinamide adenine dinucleotide (NAD^+^), 3,5‐dinitrosalicylic acid (DNS), sodium hydroxide, potassium sodium tartrate, phenol, sodium sulfite, and sodium nitrate hexahydrate were provided by Sinopharm (Shanghai, China). Kanamycin, isopropyl‐β‐d‐thiogalactoside (IPTG), and dopamine hydrochloride were all purchased from Sigma (St. Louis, MO, USA). The Ni‐NTA resin was supplied by Nano‐Micro Co. Ltd (Suzhou, China). Unless otherwise stated, all chemicals and reagents were commercial analytical‐ or biological‐grade.

### 2.2 Synthesis and characterization of NiFe_2_O_4_ magnetic nanoparticles (NF‐MNPs)

The NF‐MNPs were synthesized by the hydrothermal method [22]. Briefly, 0.73 g nickel nitrate (2.5 mM) and 2.02 g ferric nitrate (5 mM) was dissolved entirely in 50 mL distilled water. Subsequently, sodium hydroxide solution (6 M) was further added into the above suspension drop by drop until the mixture's pH was adjusted to 12. The resulting suspension was stirred vigorously for 30 min on a magnetic stirrer at room temperature. The suspension was transferred to a Teflon‐lined autoclave and heated at 180℃ for 12 h. After cooled to room temperature, the precipitate was separated by an extra magnetic field and washed several times with ethanol and distilled water separately. Finally, the prepared nanoparticles were dried in a vacuum oven at 60℃ for 6 h.

The prepared NF‐MNPs were characterized by FT‐IR (Nicolet Avatar 370 DTGS) and microscope (Olympus CH20).

### 2.3 Enzyme assay

The activities of free and immobilized His‐tagged GluDH were determined using glucose as a substrate. In brief, about 0.3 mL cell lysis contained His‐tagged GluDH or 20 mg immobilized GluDH was mixed with 1.3 or 1.6 mL Tris‐HCl buffer (pH 8.0, 50 mM), respectively. Then, 0.2 mL NAD^+^ solution (10 mM) and 0.2 mL glucose solution (100 mM) were added to the above mixture, incubating 5 min in the water bath at 50℃. Subsequently, the reaction was terminated by adding the mixture into a tube containing 3 mL DNS regent (1 L aqueous solution containing 6.3 g DNS, 262 mL NaOH of 2 M, 182 g potassium sodium tartrate) boiling immediately for 5 min. The absorbance value was measured at 540 nm using an ultraviolet spectrophotometer (UV), A unit of enzyme activity was defined as the amount of enzyme required to consume 1 μM glucose per minute.

The enzyme activity recovery and protein loading were calculated according to the following equations:

(1) $$\begin{array}{ccc} & & Activity\;recovery\;\left(\%\right)=\\ & & \frac{Total\;activity\;of\;immobilized\;GluDH}{Total\;activity\;of\;cell\;lysis\;added\;in\;reaction\;system} \end{array}$$

(2) $$\begin{array}{ccc} & & Protein\;loading\;\left(\mu g/mg\right)=\\ & & \;\frac{Total\;protein\;mass-supernatant\;protein\;mass}{Mass\;of\;nanoparticles\;for\;immobization} \end{array}$$

### 2.4 Preparation of cell lysate containing recombinant His‐tagged glucose dehydrogenase

*E. coli* BL21 (DE3) cells contained His‐tagged glucose dehydrogenase (GluDH) gene were cultured in sterilized LB medium with 50 μg mL^–1^ kanamycin for 2.5 h at 37℃ and 200 rpm. IPTG was added in the medium to a final concentration of 0.2 mM when OD600 of the mixture was 0.6–0.8. The cells were continuously incubated at 15℃ for 6 h to induce the protein overexpression. They were harvested through centrifugation at 5000 × *g* for 10 min and rinsed with binding buffer (0.05 M Tris‐HCl, pH 8.0) at 4°C; then resuspended with 5 mL lysis buffer and disrupted with sonication at 4°C. Subsequently, the supernatant was obtained by centrifuging at 8000 × *g* for 10 min to obtain the cell lysate containing His‐tagged GluDH.

### 2.5 One‐step immobilization and purification of His‐tagged GluDH with NF‐MNPs

The prepared NF‐MNPs were used as supports to purify and immobilize His‐tagged GluDH from the crude cell lysis in one step. The effects of enzyme‐supports ratio, pH, temperature, and immobilization time on the immobilization were investigated. Typically, about 20 mg NF‐MNPs were incubated with 1.0 mL clarified cell lysate with rotational shaking. Subsequently, the nanoparticles were separated with an extra magnetic field rinsed with Tris‐HCl buffer (0.05 M, pH 8.0) twice to remove nonspecific adsorbed protein. The effect of enzyme‐support ratio was determined from 0.01 to 0.16 mg mg^–1^ in pH 8.0 Tris‐HCl buffer at 30℃ for 2 h. The immobilization pH was in the range of 5.0 to 10.0, when the enzyme‐support ratio, temperature, and immobilization time were 0.13 mg mg^–1^, 30℃, and 2 h. The effect of temperature on immobilization was varied from 4 to 50℃ when the conditions were 0.13 mg mg^–1^ enzyme‐support ratio, pH 8.0 Tris‐HCl buffer, and 2 h immobilization time. The protein concentration in the supernatant was determined with the Bradford assay [42]. The activity recovery and protein loading were calculated. Each experiment was repeated triplicate, and the standard deviation was calculated accordingly.

### 2.6 Characterization of the immobilized GluDH

The relative activities of free and immobilized His‐tagged GluDH were measured at the temperature range from 25 to 60℃, pH range from 4.0 to 10.6, respectively. The buffers used were Citrate buffer (pH 4.0–6.0), Sodium phosphate (pH 6.0 and 7.0), Tris‐HCl (pH 7.0–9.0), and Glycine‐NaOH (pH 9.0–10.6). Furthermore, the effect of different metal ions and surfactants on the activities of free and immobilized His‐tagged GluDH was examined. The concentration of Ca^2+^, Zn^2+^, Mn^2+^, Co^2+^, Mg^2+^, Li^+^, and EDTA were 5 mM, and that of the Tween 80 and Triton X‐100 were 1% (w/w), respectively.

The pH‐stability of the free and NF‐MNPs immobilized His‐tagged GluDH was tested by comparing the initial enzymatic activity and residual activity of free and immobilized enzyme after 24 h incubation in verifying pH buffer range from 4.0 to 10.0. Thermostability of free and immobilized His‐tagged GluDH was investigated by incubating them in 50 mM Tris‐HCl buffer (pH 8.0) at a specified temperature between 25 and 50℃. The reusability of immobilized His‐tagged GluDH was evaluated by repeated utilization of the immobilized His‐tagged GluDH to catalyze d‐glucose to gluconolactone at 45℃ and pH 8.0. The activity obtained in each round was compared with the initial activity to calculate the relative activity.

To study the binding specificity between the NF‐MNPs and His‐tagged GluDH, the commercial Ni‐NTA resin was also employed as a support to purify the His‐tagged GluDH from crude cell lysis. Specifically, about 20 mg NF‐MNPs were added in the 5 mL Eppendorf tube containing 2 mL cell lysis solution, and the mixture was incubated at 30℃ for 2 h. For contrast, 0.1 g wet weight commercial Ni‐NTA resin was mixed with 2 mL cell lysis and incubated at 4℃ for 2 h. Then, the immobilized GluDH were separated with the assistance of a magnetic field and washed twice with Tris‐HCl buffer. Subsequently, the GluDH was eluted with 0.5 mL elution buffer containing varied concentrations of imidazole from 20 to 500 mM in turn. Finally, the collected samples were analyzed by sodium dodecyl sulfate‐polyacrylamide gel electrophoresis (SDS‐PAGE) to verify the binding specificity between the NF‐MNPs and His‐tagged GluDH.

The kinetic parameters for free‐state and immobilized GluDH were determined by measuring the activity in different substrate concentrations in the range of 0–40 mM. The *V*_max_ and *K_m_* values were calculated by nonlinear regression with the fitting of the Michaelis–Menten equation.

## 3 RESULTS AND DISCUSSION

### 3.1 Characterization of the prepared NF‐MNPs

**FIGURE 2:**
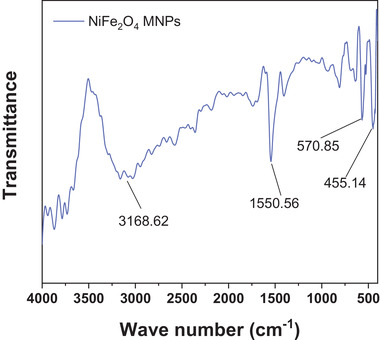
FT‐IR spectra of NiFe_2_O_4_ magnetic nanoparticles

**FIGURE 3:**
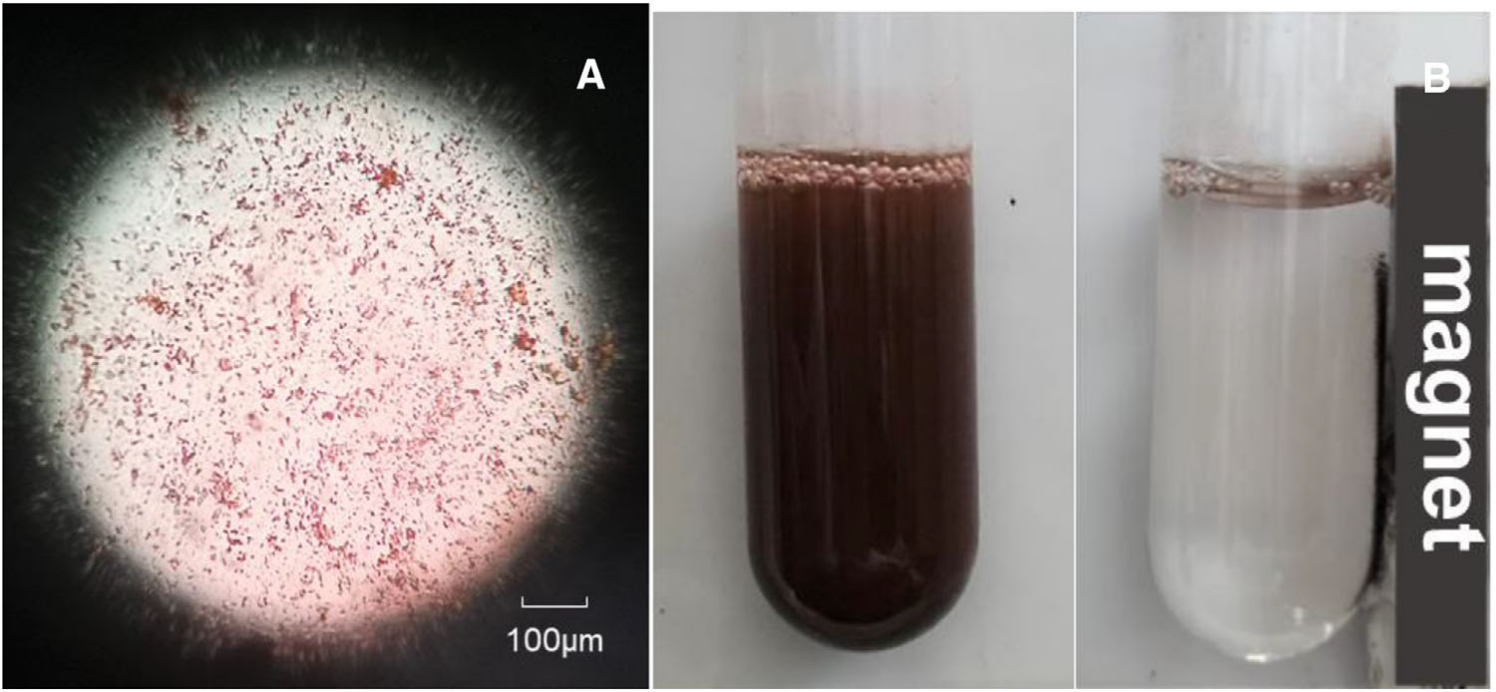
Morphology and magnetic performance of NiFe_2_O_4_ nanoparticles

The FT‐IR absorption spectrum in the range of 4000–400 cm^–1^ of as‐prepared NF‐MNPs is given in Figure 2. The adsorption bands around 570.85 and 455.14 cm^–1^ are associated with Fe–O and Ni–O stretching vibrations, two characteristic peaks of nickel ferrites prepared in this work. The peaks that appeared at 1550.56 and 3168.62 cm^−1^ are due to O–H stretching and bending vibrations of H_2_O molecules absorbed by nanoparticles, respectively [25]. The morphology features of NF‐MNPs were observed in Figure 3A, that the particle size was ranged from 30–80 nm. From Figure 3B, the nanoparticles can be homogeneously dispersed in buffer solution (left), and they can be separated easily by application of an external magnetic field in 10 s (right).

### 3.2 Optimization of the immobilization conditions

**FIGURE 4:**
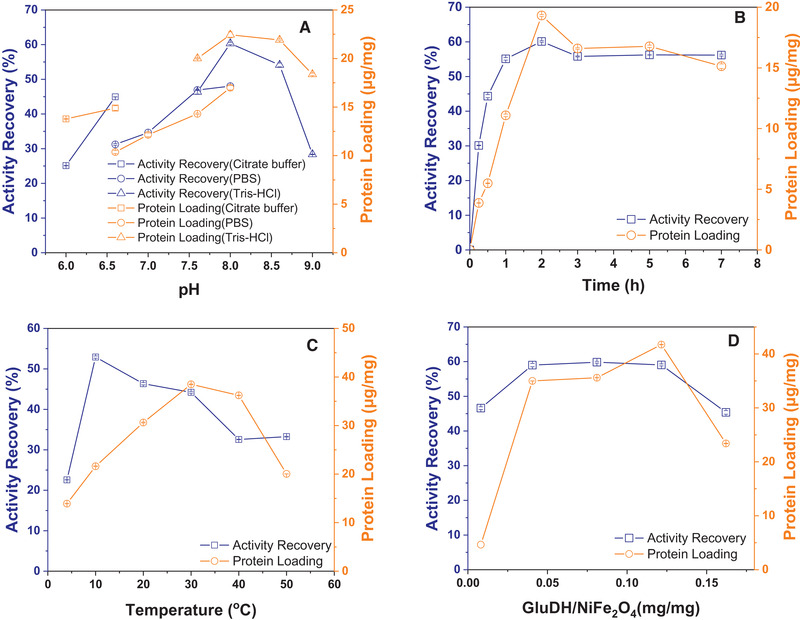
The effects of buffer pH (A), time (B), temperature (C), and the ratio of enzyme and support (D) on the activity recovery and protein loading of the immobilization and purification

The immobilization optimization is necessary for one‐step immobilization and purification of His‐tagged GluDH to obtain high activity recovery and protein binding. The effect of pH, immobilization time, temperature, and the enzyme‐supports ratio on the immobilization were studied respectively with mono‐factorial experiments. The results are shown in Figure 4A–D. The effects of pH (6.0–9.0) on recovery activity and protein loading after the incubation process were studied given in Figure 4A. The activity recovery increased by 25.23%, and protein loading was 22.44 μg mg^–1^ support when pH values varying from 6.0 to 8.0. And the maximum values of activity recovery and protein loading were achieved at pH 8.0, and there was a significant advantage that the activity recovery and protein loading were all higher in Tris‐HCl buffer than that in PBS buffer. The highest activity recovery and protein loadings were 60.34% and 22.44 μg mg^–1^ support at pH 8.0 in Tris‐HCl buffer (50 mM). Besides, when the pH value increased to 9.0, the activity recovery and protein loading reduced to 28.36% and 18.37 μg mg^–1^ support, respectively. During the immobilization process, the enzyme exhibited low activity recovery, probably because of less effective interaction between the enzyme and particles, which lead to the formation of a less activity enzyme‐particle composition [43].

The effect of immobilization time on the immobilization was determined, and the result is shown in Figure 4B. The activity recovery and protein loading increased to the highest value of 60.11% and 19.31 μg mg^–1^ support at 2 h, respectively. It is probably that the Ni^2+^ available for binding of enzyme molecules on nanocarriers tends to be saturated. Moreover, the aggregation of nanoparticles increases the particle's size and decreases the immobilization capacity of proteins. The effect of temperature on the immobilization of His‐tagged GluDH was estimated. Figure 4C shows that the highest activity recovery of 52.93% was obtained at 10℃, while the maximum value of protein loading of 38.50 μg mg^–1^ support reached 30℃. When the temperature is raised from 10 to 30℃, the activity recovery gradually declined by 8.7%, and it may be the rising temperature to change the enzyme's structure. When the temperature increased from 4 to 30℃, the collision was promoted between enzyme molecules and carriers, resulting in improved binding efficiency. Figure 4D shows that the activity recovery and protein loading reached the maximum value of 59% and 41.73 μg mg^–1^ support when the enzyme‐support ratio was 0.13 mg mg^–1^.

After considering all the factors, the proper immobilization conditions were pH 8.0 Tris‐HCl buffer, the temperature of 30℃, immobilization time of 2 h, and the enzyme‐support ratio of 0.13 mg mg^–1^. The highest activity recovery and protein bindings were achieved as 71.39% and 38.50 μg mg^–1^ support at these conditions. Furthermore, the specific activities of crude lysate containing GluDH and immobilized His‐tagged GluDH were assayed to be 1.35 and 6.18 U mg^–1^, respectively.

### 3.3 Characterization of the immobilized GluDH

**FIGURE 5:**
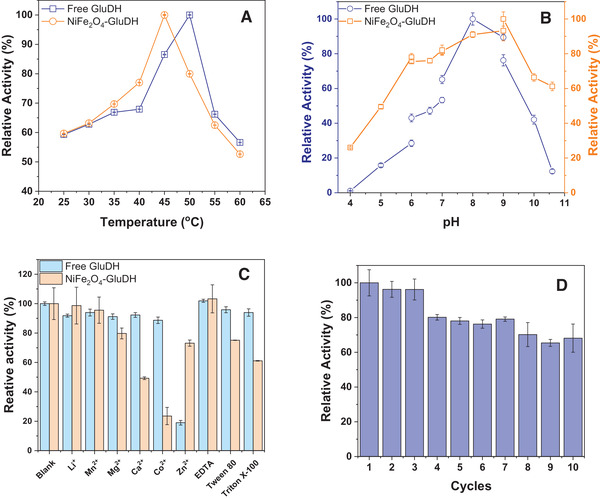
The effects of temperature (A), pH (B), metal ions, and surfactants (C) on the activity of the free and immobilized GluDH. The reusability of the immobilized GluDH (D)

The effect of temperature on the relative activity of free and immobilized His‐tagged GluDH was measured in Figure 5A. When the temperature is raised from 25 to 50℃, the relative activity of free GluDH was increased by 40.7% and attended the highest of 100%. The relative activity of immobilized GluDH was increased by 40.4% and reached the maximum at 45℃. The shift of optimal temperature might be caused by the conformation change after the immobilization. As the increasing of temperature, the relative activity of the free and immobilized GluDH decreased by 43.3 and 47.3%, respectively. When the temperature varied from 25 to 45℃, increasing temperature maybe promote the enzymatic catalytic reaction. However, it may be lead to the destruction of hydrogen bonds in the protein structure when the temperature reached 60℃, resulting in the spatial protein structure more loosely [44].

With the investigation of buffer pH, the relative activity of free and immobilized GluDH is described in Figure 5B. The relative activities of immobilized GluDH were 26.0, 49.55, and 78.02% when the pH values were 4.0, 5.0, and 6.0 in citrate buffer. While the relative activities of free GluDH were 1.10, 15.78, and 28.47%, respectively. The relative activities of the immobilized GluDH were more than 75%, while that of the free enzyme were less than 53% at pH 6.0, 6.6, and 7.0. Similarly, the relative activities of the immobilized GluDH were 66.37 and 61.21% at pH 10.0 and 11.0, respectively. However, the relative activities of free GluDH were 42.1 and 12.3% at the same pH environment. These results may ascribe the chargeability of protein and support, which affect the coordination of Ni^2+^ with the imidazole group of histidine tags and result in activity variation of the free and immobilized enzyme [45]. Furthermore, in contrast with free GluDH, the immobilized GluDH shown a wider pH tolerance range. This may be the binding between support and enzyme molecules stabilizing the complex's stereostructure and reducing the impact of environmental pH.

The effect of metal ions and surfactants on the free and immobilized GluDH is displayed in Figure 5C. The immobilized enzyme activity lost 27%, while the free enzyme lost 81% when Zn^2+^ was added. This may be on account that the Zn^2+^ had an inhibitory effect on GluDH. The immobilized GluDH activity was reduced by 21 and 51% with the addition of Mg^2+^ and Ca^2+^, respectively. After adding Co^2+^, the immobilized GluDH activity decreased by 76% of initial activity while free enzyme activity only reduced by 11%. This phenomenon may be explained by a similar affinity interaction between the imidazole group with Co^2+^ or Ni^2+^. Besides, Tween 80 and Triton X‐100 resulted in the decreased activity of immobilized GluDH by 25 and 39%, respectively.

**FIGURE 6:**
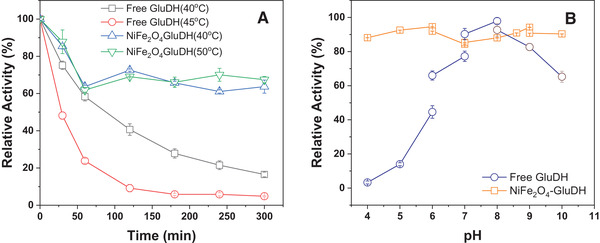
Thermostability (A) and pH stability (B) of immobilized His‐tagged GluDH

Thermostability of the free and immobilized GluDH was estimated. As shown in Figure 6A, the activity loss of immobilized GluDH was less than 27% after 120 min at 40℃, while free GluDH was more than 60%. Immobilized GluDH retained more than 60% residual activity after 300 min, while the free GluDH declined to 16.5%, the residual activity of immobilized GluDH was 3.86 times as much as that of free GliDH after 300 min at 40℃. Furthermore, the free GluDH activity decreased by 76% after 60 min retaining at 45℃, and suffered a loss of more than 91% after 120 min. However, the reduced activity of immobilized GluDH was about 35% after 300 min incubation at 50℃. The results showed that the immobilized GluDH was more stable than the free enzyme. It can be attributed to the structural stability of the NiFe_2_O_4_‐GluDH composite. The interaction of enzymes and support enhances the enzyme's stability by retarding biomolecules' free motion at an increased temperature [46].

The pH‐stability of free and immobilized GluDH was investigated, as shown in Figure 6B. The activity of the immobilized GluDH retained more than 84% of the initial activity after 24 h incubating in different pH (4.0–10.0). Simultaneously, the activity of free GluDH significantly reduced to 3.27, 13.99, and 44.58% at pH 4.0, 5.0, and 6.0, respectively. The pH‐stability of immobilized GluDH was about 27 times as much as that of free GluDH at pH 4.0. The free enzyme's residual activities were 90.24, 97.86, and 92.67% in pH 7.0, 8.0, and 9.0 of Tris‐HCl buffer, while those of the immobilized enzyme were 85.33, 88.03, and 94.19% at the same pH. When the pH value reached 10.0, the residual activity of the immobilized GluDH was 90.33%, while that of free GluDH was 65.24%. These results indicated that the immobilized GluDH exhibited a broader working pH and a higher pH tolerance than the free enzyme.

It is fast and convenient that immobilization enzymes on magnetic materials to recycle and reuse enzymes with the applied magnetic field, and then the recycled immobilized enzyme still has good dispersion in the buffer and high catalysis efficiency. The reusability of immobilized GluDH was determined by utilizing the acquired biocatalysts to convert glucose to gluconolactone for up to 10 cycles. According to Figure 5D, the immobilized enzyme can retain about 65% of its initial activity after 10 consecutive utilization. Herein, the reduction in residual activity of immobilized GluDH on NF‐MNPs after 10 cycles maybe due to the leaching [47]. The result obtained by this work has an advantage over the report regarding immobilization of GluDH on hierarchically porous silica support (MM‐SBA‐15), and after 10 cycles of use, the remaining activity of the immobilized enzyme was only 34% [48].

To evaluate the binding specificity between His‐tagged GluDH and the supports, the prepared NF‐MNPs and the commercial Ni‐NTA resin were compared for immobilization and purification of His‐tagged GluDH from crude cell lysate for comparison. The results shown in Figure S1 indicate that the affinity between NF‐MNPs and His‐tagged GluDH was highly specific and while the specifical affinity between the commercial resin and His‐tagged GluDH was relatively low. Herein, the NF‐MNPs were better for purification and immobilization of His‐tagged recombinant GluDH in industrial applications, comparing that of the commercial Ni‐NTA resin.

The Michaelis–Menten model was used to calculate the *V*_max_ and *K_m_* values of free‐state GluDH and GluDH immobilized on NF‐MNPs. The *V*_max_ and *K_m_* values of the free enzyme were 163.5 U mg^–1^ and 4.5 mM, respectively. After immobilization, the *V*_max_ was 24.3 U mg^–1^, there was about a 6.7‐fold decrease in *V*_max_ value compared with the free enzyme. The increase of *K_m_* value from 4.5 to 5.2 mM may be due to the steric hindrance of support after immobilization (Table 1).

**TABLE 1:** The kinetic parameters for free and immobilized GluDH

	*K*m (mM)	*V*max (U mg^–1^)
Free GluDH	4.5	163.5
NF‐MNPs‐GluDH	5.2	24.3

## 4 CONCLUDING REMARKS

In this work, nickel ferrite was prepared throughout the hydrothermal method and displayed high specific binding of His‐tagged glucose dehydrogenase. It was used for one‐step specifical affinity immobilization and purification of His‐tag GluDH. The highest activity recovery and protein loadings were 71.39% and 38.50 μg mg^–1^ support, respectively. The immobilized GluDH exhibited higher thermal stability at 40 and 50℃ after 300 min (60% residual activity) than the free enzyme, which indicated that the immobilization increased the thermal tolerance of GluDH. Immobilized GluDH retained over 65% initial activity after 10 cycles, and it is significant for recovery and reuse of GluDH. The His‐tagged GluDH can be easily immobilized and purified from crude cell lysate in one step due to the high specific affinity between NiFe_2_O_4_ carriers and His‐tagged GluDH.

## CONFLICT OF INTEREST

The authors have declared no conflict of interest.

## Supporting information

Supporting information.

## Data Availability

The data that support the findings of this study are available on request from the corresponding author.
