# Supplementary material for: One‐step selective affinity purification and immobilization of His‐tagged enzyme by recyclable magnetic nanoparticles
Source: Eng Life Sci. 2021 May 4;21(6):364–73. doi: 10.1002/elsc.202000093 (PMC8182278; doi:10.1002/elsc.202000093)
Supplement: Supplementary file 1 — Supporting information. [file ELSC-21-364-s001.pdf]

Research Article

Title:

One-step selective affinity purification and immobilization of His-tagged enzyme by recyclable magnetic nanoparticles

[1]Li-Jian Zhou[#], [2]Rui-Fang Li[#], [2]Xue-Yong Li, [2]Ye-Wang Zhang*

Supporting information

To evaluate the specificity of binding between His-tagged GluDH and supports, the prepared $NiFe_2O_4$ magnetic nanoparticles and the commercial Ni-NTA resin were all used for immobilization and purification of His-tagged GluDH from crude cell lysate for comparison. In Figure S1(a), a single band appeared at 27 kDa on gel when the enzyme-NF-MNPs complex was eluted with 300, 400, and 500 mM imidazole solution. Several bands can be observed between 25 to 50 kDa on gel when the commercial resin binding enzyme complex was eluted with 20, 50, 100, 200, and 300 mM imidazole solutions. According to Figure S1(b), no band was observed on lanes 3' and 4' while many bands appeared on lane 3. This phenomenon indicated that no miscellaneous protein was bound on NF-MNPs; many miscellaneous proteins were adsorbed on commercial products. These results indicated that the affinity between NF-MNPs and His-tagged GluDH was highly specific, and while the specifical affinity between the commercial resin and His-tagged GluDH was relatively low. Herein, the NF-MNPs was better for purification and immobilization of His-tagged recombinant GluDH in industrial applications, comparing that of the commercially Ni-NTA resin product.

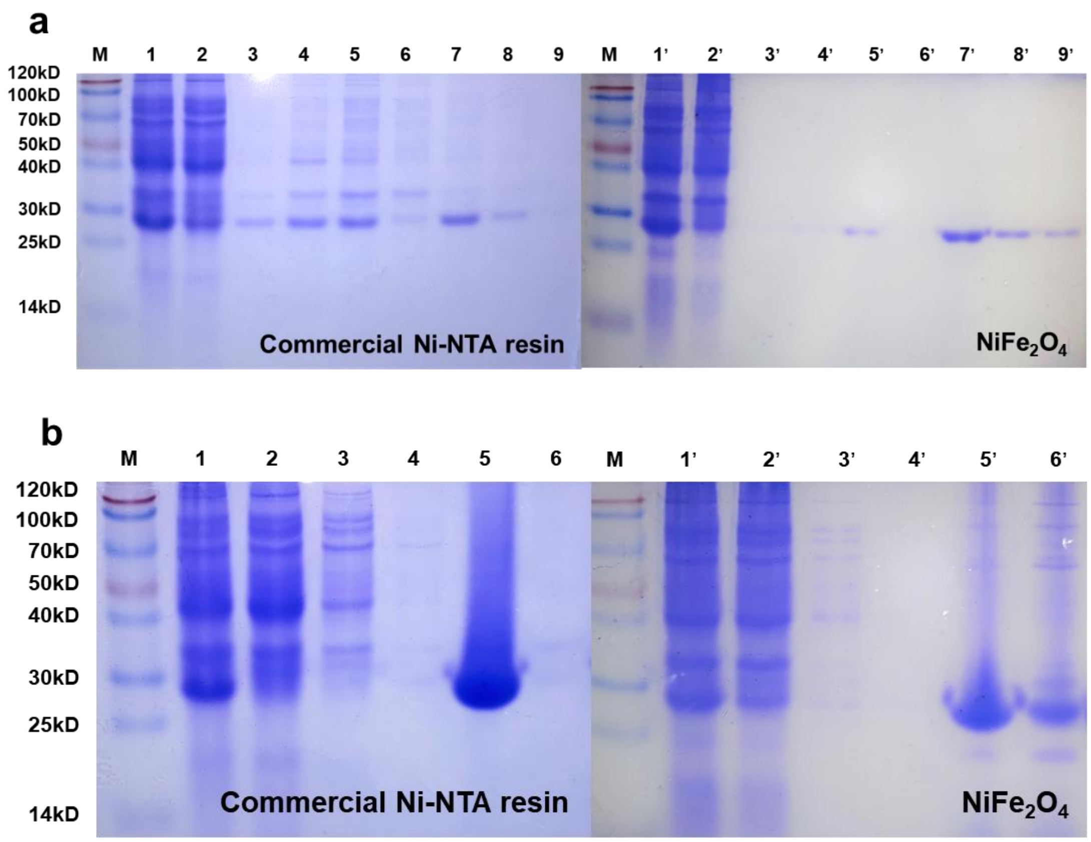

Figure S1 SDS-PAGE analysis of the specific binding His-tagged GluDH on $NiFe_2O_4$ nanoparticles and commercial Ni-NTA resin. a: lane M: Marker; lane 1-crude cell lysis; lane 2-supernatant; lanes 3~9-supernatant after eluting with different concentrations.
